# Transdifferentiation: a new promise for neurodegenerative diseases

**DOI:** 10.1038/s41419-018-0891-4

**Published:** 2018-08-06

**Authors:** Cristiana Mollinari, Jian Zhao, Leonardo Lupacchini, Enrico Garaci, Daniela Merlo, Gang Pei

**Affiliations:** 1 Department of Neuroscience, Istituto Superiore di Sanità, Viale Regina Elena 299, 00161 Rome, Italy; 2 Institute of Translational Pharmacology, National Research Council, Via Fosso del Cavaliere 100, 00133 Rome, Italy; 3 Translational Medical Center for Stem Cell Therapy, Shanghai East Hospital, School of Medicine, 1800 Yuntai Road, Shanghai, China; 4 School of Life Science and Technology, Tongji University, Shanghai, 200092 China; 5 IRCCS San Raffaele Pisana, Via di Val Cannuta 247, 00166 Rome, Italy; 6 University San Raffaele, Via di Val Cannuta 247, 00166 Rome, Italy; 7 State Key Laboratory of Cell Biology, CAS Center for Excellence in Molecular Cell Science, Shanghai Institute of Biochemistry and Cell Biology, Chinese Academy of Sciences, 320 Yueyang Road, Shanghai, 200031 P. R. China

## Abstract

Neurodegenerative diseases are characterized by a gradual loss of cognitive and physical functions. Medications for these disorders are limited and treat the symptoms only. There are no disease-modifying therapies available, which have been shown to slow or stop the continuing loss of neurons. Transdifferentiation, whereby somatic cells are reprogrammed into another lineage without going through an intermediate proliferative pluripotent stem cell stage, provides an alternative strategy for regenerative medicine and disease modeling. In particular, the transdifferentiation of somatic cells into specific subset of patient-specific neuronal cells offers alternative autologous cell therapeutic strategies for neurodegenerative disorders and presents a rich source of using diverse somatic cell types for relevant applications in translational, personalized medicine, as well as human mechanistic study, new drug-target identification, and novel drug screening systems. Here, we provide a comprehensive overview of the recent development of transdifferentiation research, with particular attention to chemical-induced transdifferentiation and perspectives for modeling and treatment of neurodegenerative diseases.

## Facts

- There are no disease-modifying therapies available for neurodegenerative diseases.
- Adult somatic cells can be reprogrammed towards a neuronal cell type in a process called transdifferentiation.
- Transdifferentiation can be achieved by cell-permeable small molecules without the need for viral transduction.
- Induced neural progenitor cells and neurons can be generated from patient-specific adult cells for regenerative and personalized medicine.
- In situ glial cells can be converted into neurons in vivo.

## Open Questions

- Can the efficiency of ciN generation from adult human somatic cells be improved for further translational applications?
- Can small molecules or transcription factors efficiently and safely be delivered across the brain–blood barrier to the diseased brain?
- Is it possible to set up protocols to reprogram cells towards a neurotransmitter and region-specific phenotypes?

## Introduction

Neurodegenerative diseases are incurable and debilitating conditions that result in progressive damage and death of neuronal cells, which leads to increasing loss of cognitive and physical functions. Although treatments may help alleviate some of the physical or mental symptoms associated with neurodegenerative diseases, there is currently no cure or way to slow disease progression^1^. Several factors have probably contributed to the failures in drug development, including unsuitable pre-clinical research models such as transgenic mice that do not fully recapitulate the human disease, as well as lacking of human in vitro or in vivo system can be referred to/compared with.

Due to increasing life expectancy, the number of people with neurodegenerative diseases worldwide is fast approaching 100 million, which has already resulted in huge economic hit and strain on the society. Therefore, researchers have been longing for the development of proper human disease models such as patient-specific neuronal cells and thereby pursue alternative strategies for modeling neurological diseases and for regenerative medicine.

**Fig. 1:**
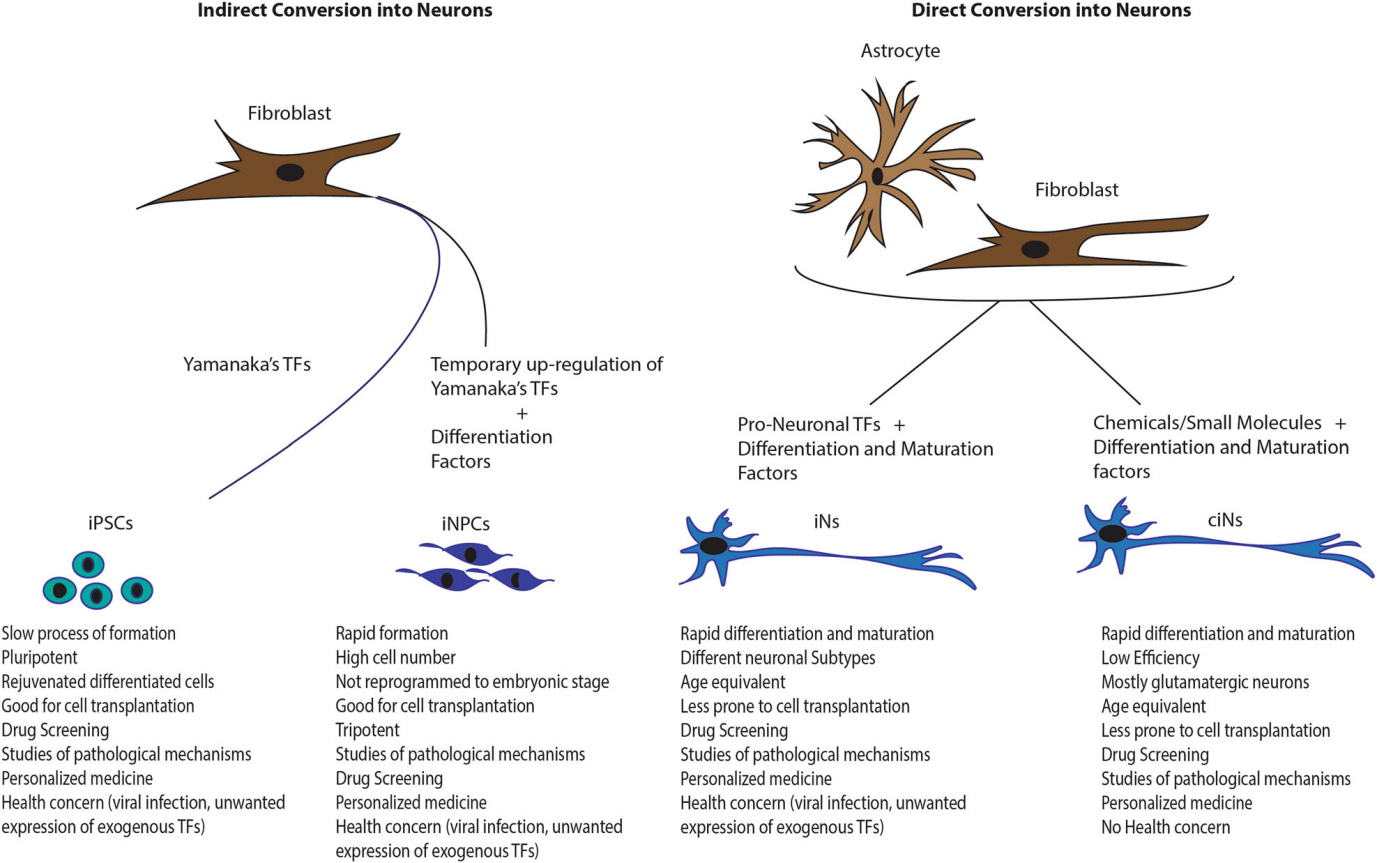
Comparison between indirect and direct conversion of somatic cells into neurons. Somatic cells can be converted into neurons either indirectly or directly. In the indirect conversion technology, fibroblasts can be first converted into iPSCs, by over-expressing Yamanaka’s TFs or into iNPCs, by temporarily over-expressing Yamanaka’s TFs in the presence of specific exogenous differentiation factors. In turn, iPSCs and iNPCS, when cultured in specific lineage differentiation medium, can generate neurons. However, neurons obtained from iPSCs are reprogrammed to the embryonic stage, thus loosing specific age-related and epigenetic features. In the direct conversion technology, age equivalent neurons can be obtained from astrocytes and fibroblasts either by pro-neuronal transcription factors (TFs) plus differentiation and maturation factors (induced Neurons, iNs), or by chemicals/small molecules (chemical-induced neurons, ciNs). iNs and ciNs are mature and functional neurons rapidly obtainable but with limitative regenerative capacities. Despite the multiple neuronal phenotypes, iNs present health concerns

In the last 60 years it has largely emerged that, under certain experimental conditions, adult differentiated cells may lose their tissue or germ layer-specific phenotypes and become reprogrammed into distantly related cell types^2–6^. Recent cellular models created from patient cells using induced pluripotent stem cell (iPSC) technology have provided promising tools for understanding human brain disease mechanisms^7,8^. However, the procedure for differentiating iPSC into functional neural cells is complicated, time-consuming, and is still on the way to be standardized^9,10^. Moreover, the iPSC-derived neurons transit through an embryo-like stage; therefore, the epigenetic codes imparted by aging or the disease progress might be erased or altered. In contrast, we and other researchers show that it is possible to switch the phenotype of one somatic cell type to another in a process of cellular reprogramming called transdifferentiation, with which neurons or glial cells can be directly induced from somatic cells without requiring a stem cell-like stage^11,12^, thus better retaining the signatures of their donors^13^. This conceptual difference might turn out to be very important for future comparative models of sporadic late-onset diseases. In addition, with less induction steps, direct conversion transdifferentiation approaches could be much faster and more efficient, and even safer for cell therapeutic application as the tumorigenesis possibilities linked with pluripotent stem cells could be excluded (Fig. 1).

So far, two major direct neural transdifferentiation approaches have been established: the first one points at direct reprogramming somatic cells into neurons, termed induced neurons (iNs), resulting however in a limited number of functional cells;^14–17^ the second approach aims at deriving neural precursors that are still proliferative (induced neural progenitor cells, iNPCs)^18–20^, which increases the feasibility of further applications demanding high cell numbers^13,21^.

In this review, we will focus on the most recent developments in the field of transdifferentiation and particularly on generation of both iNs and iNPCs from different types of somatic cells and their possible applications in neurodegenerative diseases.

## TF-mediated transdifferentiation towards a specific neuronal population

Since the discovery of MyoD, a key regulatory transcription factor capable of inducing many features of muscle cells in fibroblasts^22^, several other transcriptional regulators have emerged. In 2010, Wernig and colleagues^23^ made a significant discovery with the identification of transcription factors: Brn2, Ascl1, and Myt1 (collectively called BAM) that directly convert mouse embryonic fibroblasts to iNs. This method has then been successfully extended to the human system, where fetal and postnatal human fibroblasts are converted to iNs with BAM plus NeuroD1 transcription factors^24^.

From then on, independent groups have demonstrated that directly induced transdifferentiation technology is capable of converting mouse and human fibroblasts into iNs and iNPCs by transient forced expression of different combinations of transcription factors and through different methods of transfection/infection^3,14,18,23,25–27^. Many groups have successfully converted mouse and human fibroblasts into iNs^28,29^, glutamatergic iNs^24,30,31^, dopaminergic iNs^10,14,15,18,32^, motor iNs^26^, cholinergic neurons^33^, serotoninergic iNs^34,35^, with different combinations of transcription factors and culture conditions with various supplements of small molecules and growth factors^36^.

A diverse array of cell types has been assessed for the conversion efficiency to various iNs, with different combinations of transcription factors and culture conditions. For example, astrocytes have been converted to neurons using a single transcription factor such as Neurog2, Brn4, NeuroD1, Ascl1, or Dlx2 in vitro and in vivo. Moreover, Sox2 alone can reprogram astrocytes and NG2+ glia cells to iNs^37–42^.

To date, fibroblast is still the most commonly used starting cell type for transdifferentiation using TFs or chemical compounds or combination of both (Fig. 1).

For clinical and experimental use of iNs, it would be desirable to develop ways to generate neurons with neurotransmitter and region-specific phenotypes. Son et al.^26^ generated directly from fibroblasts iNs with motor neuron identity, displaying functional electrophysiological properties and capacity to connect with myotubes. After been transplanted into the developing chick spinal cord, these iNs engraft in the ventral horn of the spine with axons projecting into the ventral roots. Thus, these induced motor neurons can be further explored for dissecting the functional regulatory roles of glia on neuronal survival in the pathophysiology of amyotrophic lateral sclerosis (ALS). Indeed, when cultured with glia carrying the G93A mutation in the Superoxide dismutase (Sod1) gene, a mutation found in familial forms of ALS, the survival of motor neurons decreases regardless the motor neuron genotype, indicating that glia cells have a non-cell autonomous effect on motor neuron survival. In addition, motor neurons derived from Sod1G93A fibroblasts also show reduced survival when cultured with wild-type glia, suggesting that these induced motor neurons are useful for elucidating a autonomous and/or non-autonomous cellular effects that contribute to motor neuron degeneration in ALS.

Another clinically relevant neuronal subtype that has been under intense investigation is the group of midbrain dopaminergic (DA) neurons, which are preferentially lost in the brains of Parkinson’s disease patients^43,44^. Pfisterer et al.^15^ showed that the two transcription factors Lmx1a and Foxa2, when used in combination with the BAM pool, are capable of generating iNs expressing Nurr1, a marker of midbrain identity, and crucial enzymes in catecholamine biosynthesis such as Tyrosine Hydroxylase and Aromatic l-amino acid decarboxylase. Another report by Caiazzo et al.^14^ demonstrated the generation of mouse iNs with DA features can be achieved by forced expression of transcription factors Ascl1, Nurr1, and Lmx1a. In contrast to the BAM/Foxa2/Lmx1a iNs, the Ascl1/Nurr1/Lmx1a iNs were able to release dopamine, indicating that these Ascl1/Nurr1/Lmx1a cells possess an important functional property of DA neurons. However, iNs generated in this study do not express any regional markers specific to midbrain and display immature morphology. Therefore, only generic DA neuron have been obtained in reprogramming process by forced expression of midbrain DA neuron-specific transcription factors^15^. Sheng et al.^45^ induced DA neurons either by directly converting fibroblasts with DA lineage-specific factors combined with the two iN factors *Ascl1* and *Brn2*, or by first inducing fibroblasts into iNPCs and then differentiating them to DA neurons. With both methods, they show the induced DA neurons possess functional membrane properties in vitro and in vivo^45^. Moreover, Jiang et al.^46^ showed that suppression of p53 or extracellular microenvironment that lead to cell cycle arrest at the G1 phase markedly increases the transdifferentiation efficiency of human fibroblasts to DA iNs by expression of Ascl1, Nurr1, Lmx1a, and miR124. The DA iNs express markers for midbrain DA neurons and possess active dopaminergic transmission. Colasante et al.^47^ identified a combination of five TFs (*Foxg1*, *Sox2*, *Ascl1*, *Dlx5*, and *Lhx6*), reducible to four (being *Dlx5* dispensable), which are able to convert mouse fibroblasts into GABAergic iNs with high efficiency and in relatively short time. Moreover, upon being grafted into mouse hippocampus, GABA-iNs survived, matured, integrated into host circuitry, and triggered inhibition of host granule neuron activity.

In general, the transdifferentiation process contains two stages, the conversion stage and the following maturation stage. Human iNs show a slower maturation process appear less plastic and have a higher epigenetic “hurdle” for reprogramming as compared with that induced from mouse cells^3,48^. Standardized protocols with high transdifferentiation efficiency are desirable for generating neurons with neurotransmitter and region-specific phenotypes for research use or potential clinical application. Many studies have shown that hypoxia which activates transcriptional factors including hypoxia-inducible factors promotes not only cell reprogramming but also the direct conversion of various cell types to neurons. For example, the conversion efficiency of human fibroblasts to Microtubule Associated Protein 2 (MAP2) positive neurons by BAM and NeuroD1 is increased by 2.4 folds under hypoxia condition^49^. Other studies have shown that hypoxia enhance the transdifferentiation efficiency of human fibroblasts to induced DA neurons^46^ and induced serotonergic neurons^35^.

Collectively, accelerating progress in developing more efficient transdifferentiation protocols paves new ways for mechanistic studies of complex diseases, as well as potential cell-replacement therapies.

## Chemical induced neurons

In almost all available protocols, small molecules have been applied to enhance the reprogramming or transdifferentiation efficiency along with tightly controlled expression of ectopic genes as well as further reduction of the number of TFs^28,50^. Cell-permeable small molecules have been shown to facilitate cell reprogramming^28,33,51,52^ or promote neural differentiation^53^. Moreover, studies show that small molecules alone can reprogram fibroblasts into iPSCs^54^, neuroprogenitor cells (NPCs)^55^, or directly into neurons^16,52^.

The finding that combinations of small-molecule compounds can directly convert various cell types to neurons without the need for transcription factors offers more feasibility to standardize the induction protocols as well as neuronal cell production for further investigation or application with less consideration on genome modification as compared to TF-based method (Fig. 1). Particularly, human chemical iNs (hciNs) provides an avenue for characterize human neuronal function in cultures, which may enable the discovery of more specific features of the human pathology as well as validation of current animal disease models.

Dozens of compounds have been used in various combinations to achieve somatic cell reprogramming or transdifferentiation. Research groups have been keen on improving the induction efficiency as well as understanding the underlying mechanism, which in turn will lead to further expedite the progress of reprogramming and transdifferentiation. Studies have shown compounds categorized as epigenetic modulators, MET (mesenchymal to epithelial transition)/EMT (epithelial to mesenchymal transition) modulators, regulators of metabolism, compounds promoting self-renewal of embryonic stem cells (ESCs), and etc., are required for reprogramming and transdifferentiation. Generally, histone deacetylase (HDAC) inhibitors and/or DNA/Histone methyltransferase inhibitors, are indispensable to overcome the epigenetic barrier between different types of cells. Then, compounds such as Wnt pathway activators and the transforming growth factor (TGF)-β pathway inhibitors that suppress the characteristics of the starting cells are commonly used. Indeed, CHIR99021, which activate the Wnt pathway by blocking Glycogen Synthase Kinase 3 (GSK3), is the most commonly used compound in all chemical induction systems. Moreover, compounds that induce the characteristics of the designated cells are required. For ciN induction, compounds such as ISX9 and Dorsomorphin that can promote neuronal specification or maturation have been applied^16,52,56^.

Among the chemicals used in reprogramming, valproic acid (VPA, an HDAC inhibitor), CHIR99021 (a GSK3 inhibitor), and RepSox (a TGFβ Receptor-1 inhibitor), namely VCR, are most commonly used as the core reprogramming chemicals to promote the initiation of cell fate transition. They are applied in the induction of chemical iNPCs (ciNPCs)^55,57^, chemical iNs (ciNs)^16,52^ from fibroblasts, where VPA may facilitate global transcriptional changes, and CHIR99021 may promote mesenchymal to epithelial transition. With favorable culture conditions, these activated cells can then be induced to generate various functional neuronal cells.

**Fig. 2:**
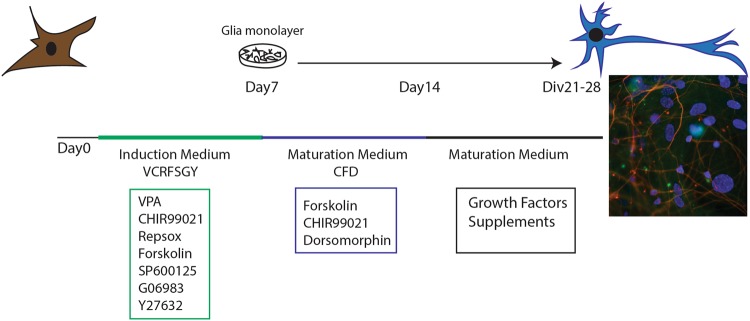
Chemical reprogramming of fibroblasts to neurons. A totally chemical approach can be used to direct reprogram fibroblasts to functional human neurons that retain the patient-specific signature such as age, epigenetic information, and pathological features. The fluorescence image in the scheme shows human ciNs, obtained from fibroblasts, labeled with antibodies against MAP2 (green), Beta III Tubulin (red), and nuclei counterstained with Hoechst (blue)

Recently, two research groups reported simultaneously the successful generation of mouse and human neurons from fibroblasts with full-chemical approaches. Li et al.^52^ discovered that the four compounds Forskolin, ISX9, CHIR99021, and SB431542 are sufficient to induce neuronal transdifferentiation, among which the neurogenesis inducer ISX9 is necessary to activate neuron-specific genes. Moreover, they found that the BET family bromodomain inhibitor I-BET151 functions to disrupt the fibroblast-specific program, thus greatly enhances the reprogramming efficiency. Therefore, final combination is made by Forskolin, ISX9, CHIR99021, and I-BET151 for inducing transdifferentiation of mouse fibroblasts to ciNs^52^. On the other hand, our lab (Hu et al. 2015)^16^ showed that a combination of seven chemicals which contains the core chemical combination VCR (VPA, CHIR99021, and Repsox) and Forskolin, SP600125, GO6983, and Y-27632 could facilitate the human fibroblast conversion into neurons. In particular, Dorsomorphin and extra neurotropic factors (BDNF, GDNF, and NT3) were able to generate fully mature and functional hciNs (Fig. 2). Furthermore, we showed that this protocol is efficient to generate ciNs from familial Alzheimer’s disease patients harboring Aβ-related pathology^16^.

Astrocytes, which play important roles in the brain and in neurodegenerative diseases^58^, can be also converted to neuronal cells by chemical cocktails containing VCR^59,60^. Zhang and colleagues^61^ identified a cocktail capable of reprogramming human astrocytes into neurons made of nine chemical compounds: LDN193189, SB431542, TTNPB, Thiazovivin, CHIR99021, VPA, DAPT, Smoothened agonist, and Purmorphamine, that can reprogram human astrocytes into neurons when added in a stepwise manner. Interestingly, these hciNs can survive for several months and form functional synaptic networks both in vitro and in vivo^61^. In our laboratory we demonstrated that adult human astrocytes can be converted into neuronal cells by a different set of small molecules and, more importantly, when transplanted into postnatal mouse brains, these induced neuronal cells survive and become physiologically mature^60^.

## From fibroblast to induced NPCs

The population of iNs has no proliferation potential which restricts further large scale application. Besides, due to their poor survivability, mature iNs are not particularly suitable for transplantation in vivo. Therefore, despite the direct conversion strategy bypasses the pluripotent stem cell stages, and thus may avoid the potential risk of tumor formation, it might also limit the final number of cells that can be obtained hence unmeet the requisite for cell-replacement-based therapies or drug screening. For these reasons, it would be desirable to induce expandable NPCs directly from fibroblasts. Ding Kim and colleagues successfully converted mouse fibroblasts to induced NPCs^19^. In this study, the protocol for reprogramming was identical to the iPSC reprogramming TF protocol, with the exception that the factors were induced only for a short time and the cells were then exposed to media favoring the growth of NPCs (Fig. 1). After optimization of timing and culture conditions, colonies that closely resembled neural rosette cells appear and express several relevant markers. Indeed, upon spontaneous differentiation, these iNPCs can give rise to multiple neuronal subtypes and astrocytic cells, indicating that the iNPCs are at least bipotential neural precursor cells.

From then on, many groups reported the generation of iNPCs using neural lineage-specific TFs. These iNPCs are multipotent and can differentiate into functional neurons, astrocytes, and oligodendrocytes both in vitro and in vivo^20,62–66^. These iNPC induction protocols generally relies on retroviral and lentiviral expression systems, thus causing concerns regarding the genomic integration of viral DNA. In order to improve the safety of iNPCs the researchers have developed protocols using either non-integrating Sendai virus^66^ or episomal plasmids^67,68^.

Interestingly, studies show that mouse and human iNPCs can be obtained by a cell-activation signaling-directed strategy^18,66,69^ based on the transient overexpression of TFs in conjunction with lineage-specific soluble signals. A recent study showed that infection of postnatal adult human and monkey fibroblasts with Sendai virus containing the Yamanaka factors (Oct3/4, Sox2, Klf4, and c-Myc), cultured in a chemically defined medium (containing: leukemia inhibitory factor, TGF-β inhibitor SB431542, and the glycogen synthase kinase-3β (GSK-3β) inhibitor CHIR99021) allows the generation of iNPCs^66^. Zhu et al.^70^ identified a small-molecule combination of A83-01 (a TGF-β inhibitor) and CHIR99021 to reprogram Oct4/Sox2-transduced human neonatal fibroblasts into colonies expressing the human NPC marker Pax6. They also found that a combination of lysophosphatidic acid (a phospholipid derivative), Rolipram (a phosphodiesterase-4 inhibitor), and SP600125 (a c-Jun N-terminal kinase inhibitor) facilitates the reprogramming of adult human dermal fibroblasts transduced with Oct4 alone^70^. Zhu et al.^70^ also demonstrated that p53 may be a master regulator of cell reprogramming, since depletion of p53 alone can generate iNPCs, and the efficiency of this protocol is increased when Neurod2 transcription factor is expressed.

An important step forward the reprogramming protocols has been achieved by us^55^. In this study we reported that, under physiologically hypoxic conditions, iNPCs can be generated from mouse embryonic fibroblasts using only the chemical core cocktail VCR. In addition, we identified alternative cocktails of molecules that, in combination with inhibitors of histone deacetylation, glycogen synthase kinase, and TGF-β pathway, have similar NPC induction effects^55^. Recently, Zhang et al.^57^ reported an efficient way to transdifferentiate mouse fibroblasts into iNPCs using a cocktail of nine different chemical components.

## Applications of transdifferentiation protocols in neurodegenerative diseases

Current therapies for neurodegenerative diseases are restricted to controlling symptoms. At present, there is no effective treatment to prevent or retard the clinical progression of these diseases. In fact, the mechanisms underlying neurodegeneration are poorly understood, thus making the target-based drug screening strategies rather difficult.

The possibility to obtain patient-specific iNs would be an important source for proteomic and transcriptomic studies that may help identifying sets of molecular signatures for the neurodegenerative disease. In addition, these patient-specific cells represent a powerful tool for personalized drug tests. Finally, direct conversion to obtain patient-specific neurons can eventually allow personal medicine and the development of autologous cell types for cell therapy in neurodegenerative disease such as ALS, Alzheimer's Disease (AD), and Parkinson's Disease (PD)^71–73^.

With the realization of inducing easily accessible cells, such as skin fibroblasts or peripheral blood mononuclear cells, to inaccessible cells that are lost in the degenerative diseases (e.g. midbrain DA neurons in PD), it becomes possible to generate cells in vitro that are increasingly similar to their in vivo counterparts and then apply these induced cells for transplantation. Several studies are exploiting transdifferentiation as a possible tool to fight neuronal loss in brain. It has been shown that transplantation of mouse DA iNs in 6-OHDA-lesioned mice restores locomotor deficits^18^. Similarly, mouse DA iNs transplanted in 6-OHDA-lesioned rats functionally integrate into the rat neuronal network and alleviate motor symptoms^50^.

iNPCs would be also very useful to obtain target cells for transplantation therapy, establishing disease models, and drug screening, as well as for monitoring curative effects.

For cell potential therapeutic strategies, iNPCs may be safe, having a low risk of tumorigenesis, while maintaining the capacity of self-renewal and giving rise to multiple neuronal subtypes in vitro and in vivo.

Indeed, iNPCs transplanted into the adult mouse brain survived for up to 6 months without graft overgrowths^74^. In a mouse model of multiple sclerosis, iNPCs, converted from mouse somatic skin fibroblasts, displayed significantly high intrinsic migratory features and anti-inflammatory capacity when co-cultured with lipopolysaccharide-activated macrophages. After the intracerebral injection of iNPCs, chronic experimental allergic encephalomyelitis in mice was ameliorated^75^.

Transplanted iNPCs derived from mouse fibroblasts through overexpression of TFs (Sox2, Klf4, and c-myc) into the hemispheres of neonatal myelin-deficient rat brain survived and gave rise to differentiated neural cells in vivo^76^.

iNPC cells can be also used to model neurodegenerative diseases such as ALS. Fibroblasts from ALS patients and age-matched healthy controls were converted to iNPCs by transfection with four reprogramming factors. These iNPCs show the potential to generate motor neurons and astrocytes^77^. Moreover, iNPCs transplanted into the contusion lesion site of rat spinal cord differentiate into all neural lineages, especially several subtypes of mature neurons, suggesting that this strategy holds therapeutic potential for restoration of spinal cord injury^78^.

Altogether, transdifferentiation into iNs or iNPCs, although with important limitations, opens a door for cell-based therapeutic intervention.

## Transdifferentiation in vivo

The ultimate goal of cell reprogramming is to define an innovative approach for cell therapy in human neurological diseases and determine whether cell conversion is applicable in vivo. In fact, different laboratories provide evidences that neural conversion can take place in vivo similar to what has already been shown for other organ systems^39,40,42,79–87^. Indeed, transplanted human fibroblasts and human astrocytes, which are engineered to express inducible forms of neural reprogramming genes, convert into neurons when reprogramming genes are activated after transplantation in the adult rodent brain^79^.

**Fig. 3:**
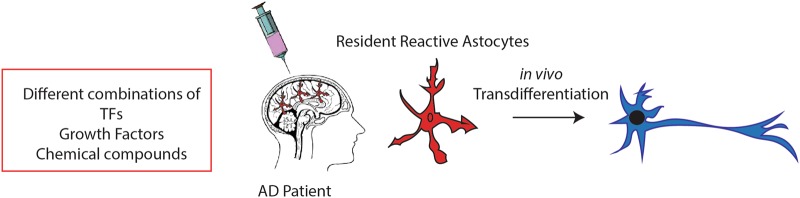
**Transdifferentiation in vivo.** In situ astrocytes can be reprogrammed in the diseased brain by using different approaches, thus representing a promising tool for regenerative medicine

Specifically, when we consider neurological diseases, an approach of converting the on-site glial cells may be a most desirable strategy that might avoid invasive cell transplantation. Interestingly, several studies have shown that iNs can be generated from endogenous mouse astrocytes that are reprogrammed by viral delivery in situ^40,42,79,82–86^, suggesting a promise for in vivo brain repair (Table 1). Similarly, overexpression of a single transcription factor, Sox2, in the injured adult spinal cord, directly transformed resident astrocytes into Doublecortin-positive neuroblasts^87^. This particular ability to convert non-neuronal cells into subtype-specific neurons directly in the diseased brain opens up novel avenues for brain repair using on-site brain cells as a starting material for induction of cell transdifferentiation (Fig. 3).

**Table 1:** In vivo reprogramming into neurons and cellular integration in the brain

Cell type	Transcription factors	Chemicals	Induced neurons	References
Mouse glia	Neurog2		Immature/GABA/Glut	83
Human astrocytes	Ascl1+Brn2+Myt1		n. d.	79
Mouse astrocytes	Sox2		Immature/n.d.	86
Mouse astrocytes	NeuroD1		Glut	42
Mouse astrocytes	Sox2		Immature/GABA	87
Mouse astrocytes	Sox2		Immature/Calretinin	84
Mouse astrocytes	NeuroD1 + Ascl1 + Lmx1a + miR128		Dopaminergic	85
Mouse astrocytes	Ascl1		GABA	40
Mouse astrocytes	Neurog2 + Bcl2		Glut	82
Human astrocytes		9 molecules^a^	Glut/Chat/GABA/TH	61
Human astrocytes		6 molecules^b^	Glut/Chat	60
Mouse NG2	Sox2		GABA	39
Mouse NG2	NeuroD1		Glut/GABA	42
Mouse NG2	Ascl1 + Lmx1a + Nurr1		Glut/GABA	81

^a^LDN193189, SB431542, TTNPB, Tzv, CHIR99021, VPA, DAPT, SAG, and Purmo

^b^VPA; Chir99021; Repsox; Forskolin; i-Bet151; ISX9

## Present limitations and future perspectives

The realization of generation of neural cell by somatic cell transdifferentiation using combinations of transcription factors and/or chemical compounds has opened up new opportunities for disease modeling and cell therapy. Clearly, transdifferentiation achieved by transgene-free or chemical-only approaches may provide alternative safer strategies for the generation of neuronal cells. Although a growing number of studies show that transdifferentiation can be achieved by small molecules alone, more investigation is required to improve the efficiency and reduce the induction variation. At the moment, future studies are needed to improve ciN generation from adult human fibroblasts since the current low efficiency represents an obstacle for further translational applications. In this respect, the microenvironment provided by 3D culture may facilitate chemical reprogramming. With present protocol, a purification step is requisite as the cellular product is not pure, but contains a mixture of pluripotent cells, cells with different degrees of differentiation, and even unmodified cells. Thus, future research should attempt to optimize the conditions for a more complete cell transdifferentiation. Further, a better understanding of the detailed mechanisms during transdifferentiation processes can also help to improve the induction efficiency as well as the cell maturation with desirable biophysical functions. Attentive studies to evaluate the genomic integrity of the cells generated with transdifferentiation will also be crucial. Another challenge ahead is how to effectively deliver small molecules or transcription factors across the brain–blood barrier to the injured or diseased brain. Hopefully, in the near future, the chemical strategy may provide alternative regenerative medicine in addition to the cell-replacement therapy for neurodegenerative diseases.
